# Factors predicting different times for brushing teeth during the day: multilevel analyses

**DOI:** 10.1186/s12903-023-03555-1

**Published:** 2023-11-24

**Authors:** Hwa-Young Lee, Nam-Hee Kim, Jin-Young Jeong, Sun-Jung Shin, Hee-Jung Park, Ichiro Kawachi

**Affiliations:** 1 Graduate School of Public Health and Healthcare Management, The Catholic University of Korea, Seoul, South Korea; 2 Catholic Institute for Public Health and Healthcare Management, The Catholic University of Korea, Seoul, South Korea; 3 Department of Dental Hygiene, Mirae Campus, Yonsei University, Wonju, South Korea; 4 Hallym Research Institute of Clinical Epidemiology, Hallym University, Chuncheon, South Korea; 5 Department of Dental Hygiene, College of Dentistry, Gangneung Wonju National University, Gangneung, South Korea; 6 Department of Dental Hygiene, College of Health Science, Kangwon National University, Samcheok, South Korea; 7 Harvard T.H. Chan School of Public Health, Boston, USA

**Keywords:** Toothbrushing, Health behavior, Oral health, Multilevel modeling

## Abstract

**Background:**

The most effective and simple intervention for preventing oral disease is toothbrushing. However, there is substantial variation in the timing of brushing teeth during the day. We aimed to identify a comprehensive set of predictors of toothbrushing after lunch and after dinner and estimated contextual (i.e., geographic) variation in brushing behavior at different times of the day.

**Methods:**

We constructed a conceptual framework for toothbrushing by reviewing health behavior models. The main data source was the 2017 Community Health Survey. We performed a four-level random intercept logistic regression to predict toothbrushing behavior. (individual, household, Gi/Gun/Gu, and Si/Do).

**Results:**

Individuals under 30 years of age had higher likelihood of brushing after lunch, while brushing after dinner was higher among those aged 40–79 years. People engaged in service/sales, agriculture/fishing/labor/mechanics, as well as student/housewife/unemployed were 0.60, 0.41, and 0.49 times less likely to brush their teeth after lunch, respectively, compared to those working in the office, but the gap narrowed to 0.97, 0.96, 0.94 for brushing after dinner. We also found significant area-level variations in the timing of brushing.

**Conclusions:**

Different patterns in association with various factors at individual-, household- and Si/Gun/Gu-levels with toothbrushing after lunch versus toothbrushing after dinner suggests a need for tailored interventions to improve toothbrushing behavior depending on the time of day.

**Supplementary Information:**

The online version contains supplementary material available at 10.1186/s12903-023-03555-1.

## Introduction

Oral diseases are slow to progress, but once they develop, there is little chance of a natural recovery, making them irreversible. Even if the lesion is excised and treated, it can still have a long-term effect that leads to periodontal disease and early tooth extraction in middle and old age. Abundant evidence also suggests that oral diseases raise the risk of developing other chronic conditions [1–5]. According to Lalla et al. (2012), oral diseases such as dental caries, Candida infection as well as periodontal disease are associated with risk of diabetes [6]. Additionally, oral diseases also place a heavy financial burden on society. Gingivitis and periodontal disease ranked first in Korea between 2019 and 2020 for the frequency of outpatient visits [7, 8]. Outpatient dental expenses also increased 5-fold, from 1.9 trillion won in 2000 to 10 trillion won in 2019 [8].

The majority of oral diseases can be avoided by adopting oral health management behaviors [9], of which toothbrushing is the most simple, effective, and inexpensive [10, 11]. A comprehensive systematic literature review and meta-analyses demonstrated a significant inverse correlation between the frequency of brushing and the risk of diabetes, suggesting that the incidence of disease risk rises by 20% for every reduction in the frequency of toothbrushing [12].

While twice-daily brushing including, ideally after meals, is widely recommended [13, 14], the evidence-based guideline on oral health, recently issued by the Department of oral health policy in the Ministry of Health and Welfare in Korea, strongly recommends “toothbrushing at least twice a day, including before bedtime”. In 2019, Korean adults brushed their teeth 2.5 times a day on average, most frequently after breakfast (60.7%), followed by after dinner (57.1%), and lunch (51.1% or more) and before going to bed (50.2%) [15]. Although the average frequency of toothbrushing is relatively high, about 20% of population still brush their teeth less than twice a day. Further, toothbrushing behavior exhibits geographic disparities similar to other health behaviors [16]. According to the 2020 Community Health Survey (CHS) data, the gap between the lowest and the highest rate among 17 cities/provinces (Si/Do hereafter) for brushing after lunch (among adults aged 19 and older) was approximately 13.5%. There was an even bigger disparity among the 255 city/country/districts (Si/Gun/Gu hereafter)(34.2%) [16].

One thing to note here is that pattern of geographic disparity differs depending on the time of the toothbrushing during the day. According to results based on the combined data from 2015–2019 CHS, men in Seoul showed the fifth highest rate among 17 Si/Dos for brushing after lunch but the lowest rate of brushing after dinner. Conversely, males in Jeju Island had the lowest rate of brushing after lunch but the second highest rate after dinner (Figure S1). These findings suggest the possibility that there are differential predictors of toothbrushing behavior according to the time of day when it is practiced. If this is the case, interventions (e.g. health communications) could be targeted to improve the adoption of brushing at different times of the day. Programs to promote oral health so far have not taken this into account.

**Fig. 1:**
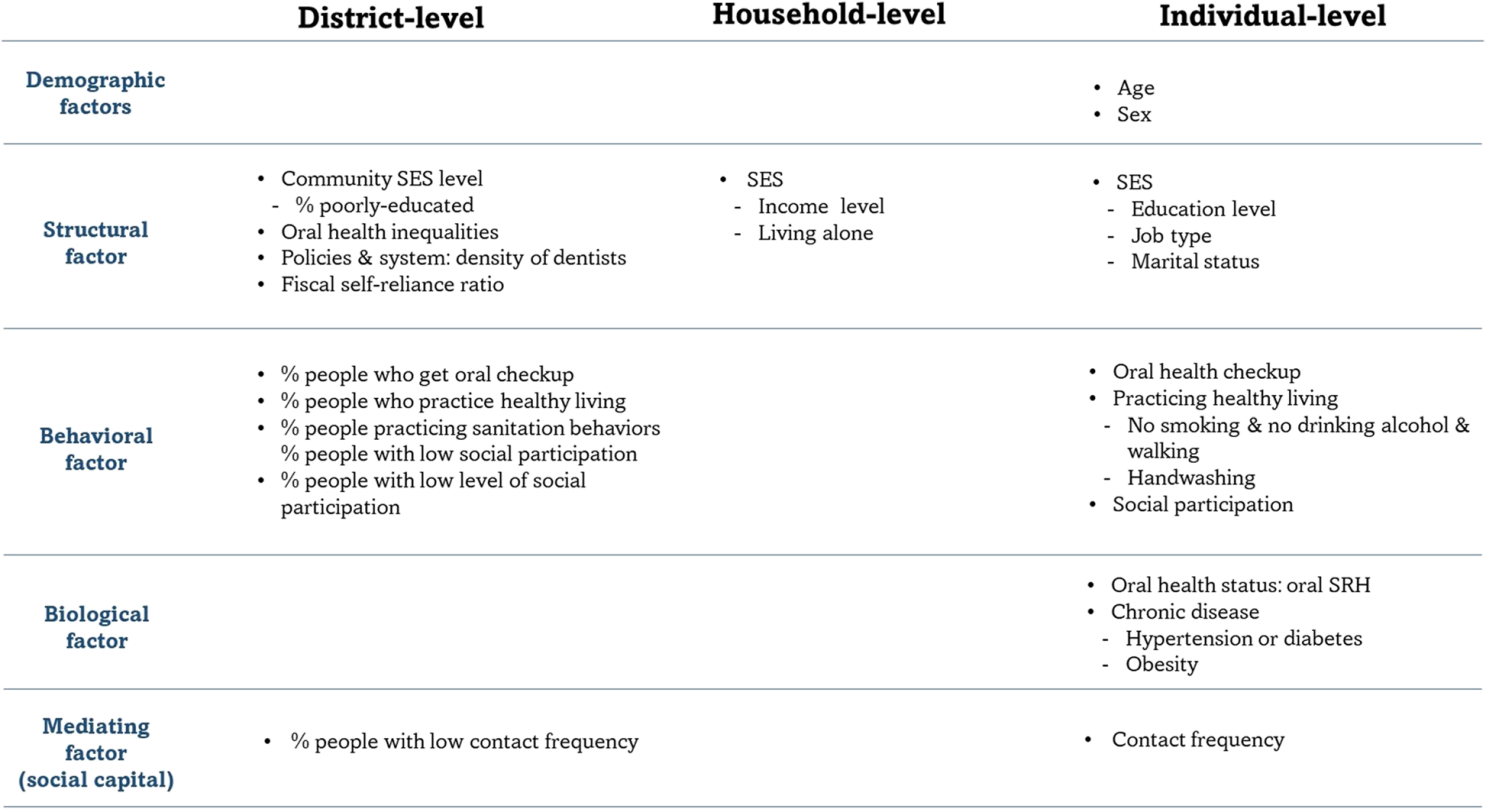
The conceptual framework for toothbrushing behavior

Few studies have examined the determinants of toothbrushing behavior and mostly focused on children [17, 18]. Additionally, they only examined individual-level factors based on single-level analysis without considering contextual factors [19], or distinguished between toothbrushing at specific times of the day [20, 21]. Accordingly, our research aims are as follows. First, we investigate what individual- and contextual- level factors predict brushing behavior after lunch versus after dinner. Second, we examine contextual (i.e., geographic) variation in brushing behavior at different times of the day. We focused on two specific time points, i.e., after lunch and after dinner based on the observation that toothbrushing rates were the lowest at these two time points, and therefore, we assumed that encouraging toothbrushing at these two time points would be effective in increasing the rate of toothbrusing two times a day including bedtime. For this, we developed a conceptual framework for toothbrushing behavior based on a review of relevant health behavior models (Fig. 1).

### Conceptual framework for toothbrushing

Health behaviors can be either goal-directed (i.e., have clear objectives or intentions such as exercising or eating to lose weight), or they can be performed habitually without any apparent purpose [22]. Behaviors in the latter category can be triggered by small cues in daily life, such as indulging in a snack while watching TV. These two aspects of behavior are not necessarily mutually exclusive. For example, an individual might consciously begin to brush after lunch with the intention of improving their oral hygiene, but the behavior can eventually become an automatic habit [14, 23, 24].

While there is an abundance of literature documenting disparities in oral health outcomes, the literature on oral health-related behaviors remains sparse. Anderson's model for medical service use [25] and Rosenstock's health belief model [26] have been the most commonly employed to describe health behavior. However, toothbrushing behavior does not fit well with either of these models. Antonovsky and Kats' integrated model of preventive oral health behavior was the best fit for describing toothbrushing behavior where they proposed three types of variables explaining preventive oral health behavior: predisposing motivation, blockage type, and conditioning type. Detailed descriptions of each variable are provided below [27].

First, people generally engage in preventive health behaviors with three goals (called “predisposing motivation”); 1) to avoid illness and improve health 2) to gain approval from others important to them, and 3) to gain self-approval. These motivations are not mutually exclusive and can reinforce each other. However, it cannot be assumed that just because someone is highly motivated by one or more of these goals, they will engage in certain health behaviors. The variable which determines whether being motivated will translate into action is the “blockage type”. Blockages can be divided into internal, which includes knowledge, anxiety or fear, versus external, which refers to the availability of resources such as time, money, or a dental care provider [27]. Finally, the variable “conditioning type” includes underlying susceptibility to illness, level of education, presence of stress, and previous negative experiences. Conditioning variables are conceptualized as moderating the effects of predisposing motivation or blockage type. Based on this model, people brush their teeth 1) to promote oral health, 2) to improve aesthetic appearance (i.e., gain approval from others), and 3) to pursue personal hygiene (self-approval).

Patrick et al. (2006) further proposed a model for mechanisms to explain oral health disparities based on macro-level factors (distal factors), community determinants at a lower level (intermediate factors), interpersonal factors that occur as a result of interactions between individuals, and lastly, individual-level factors (proximal factors) [28]. Our final conceptual framework for toothbrushing behavior was developed by merging the Patrick et al. (2006)’s model with the Antonovsky and Kats' integrated model (Fig. 1).

Willingness to promote oral health was represented by following a healthy lifestyle (walking, not drinking, and not smoking) based on assumption that those with higher awareness of general health are also mindful of their oral health. We included participation in social activities to signify seeking social approval from others. Those who are more involved in social groups interact with others more frequently and are thus more conscious about their appearance [29, 30]. As a proxy variable for self-approval, we included a variable for handwashing before meals. Handwashing before meals is not observable by others (in the same way as having clean, white teeth), and is performed to satisfy an intrinsic goal (maintaining hygiene) [31–33].

Variables related to blockage type include being up to date on oral health examination and frequency of contact with relatives/friends (a proxy for receiving information on the importance of toothbrushing). Finally, variables related to conditioning type include socioeconomic status and occupation (a proxy for the environment in which brushing takes place).

## Methods

### Data sources and study population

We performed secondary analyses using the data that were obtained from the 2017 CHS and additionally from the National Statistical Office. The CHS is conducted by Korea Disease Control and Prevention (KCDC) with the main purpose of producing health statistics at the Si/Gun/Gu level and is nationally representative.

### Study design and sampling

In CHS, a probabilistic, stratified two-stage sampling was adopted where the first stratum is Dong/Eup/Myeon (the smallest administrative unit equivalent to community) and the second is housing type (apartment vs. house). Tong/Ban/Lee (small village) were selected as the primary sampling unit (PSU) within strata with probability proportionate sampling. In the second stage, all households were identified within each of the chosen PSUs and five households within each PSU were selected using systematic random sampling. All adults aged 19 or older in sampled households were surveyed, which yielded a total of 228,381 adults in 2017 CHS. The sample size was calculated to have a sampling error of ± 3% for main health index in each community health center. More details can be found elsewhere [34].

### Outcome variables

We examined two outcome variables: toothbrushing after lunch and after dinner. The questions in CHS read: “Did you brush your teeth after lunch (or dinner) yesterday?” with three response options of ‘yes’, ‘no’ and ‘have not eaten lunch (or dinner)’. Since the preventive effect of toothbrushing from oral diseases is mainly related to toothbrushing after meals, those who skipped toothbrushing because they had not eaten were excluded from the analyses.

### Independent variables

As aforementioned, individual-, household-, and Si/Gun/Gu-level variables were selected based on the conceptual framework for toothbrushing. Individual-level factors include age and sex as demographic factors; education level, occupation, and marital status as structural factors; oral checkup, healthy lifestyle, handwashing before meal, and participation in activities as health behavioral factors; self-rated oral health, chronic diseases, and obesity as biological factors; and finally contact frequency with others as mediating factors. Household income and living alone were included as household-level variables. Si/Gun/Gu characteristics include the proportion of the population with low level of education, disparities in oral health, the density of dental clinics (or hospitals), and fiscal independence ratio as structural factors; the proportion of people who had an oral checkup, people who practice healthy lifestyle, people who wash hands before meal, and people who had low contact frequency with others as health behavioral factors; and finally the average number of social groups as a mediating factor. Details on definition and categorization of each variable are presented in Table S1.

All individual-level variables were taken from the 2017 CHS. Density of dental facilities and financial independence were extracted from the Statistics Office and the remaining Si/Gun/Gu variables were created by aggregating individual-level variables using CHS data.

### Statistical analyses

We report characteristics of the final analytic sample, using sampling weights. For main analyses, a four-level random-intercept logistic regression was adopted to reflect the random distribution of the outcome variables at the contextual level in addition to the random distribution at the individual level. A multilevel logistic regression model can be specified according to the following general structure.

$$logit\;(Pr\;(Y_{ijkl}\;=\;1))\;=\;\beta_0\;+\;X_{ijkl}\;+\;(u_{0jkl}\;+\;v_{0kl}\;+\;f_{0l})\;(\mathrm Y=\mathrm{toothbrushing}\;\mathrm{after}\;\mathrm{lunch}\;\mathrm{or}\;\mathrm{dinner})$$

Where the dependent variable Y (tooth brushing after lunch or dinner) and independent variables X (representing a vector of covariates) were each assumed to follow a multilevel data structure where individual *i*, is nested in household *j (level 2)*, Si/Gun/Gu *k(level 3)*, and Si/Do *l (level 4)* with both fixed-effect*(* β_0,_ X_*ijkl*_) and random-effects parameters (*u*_0*jkl*_, *v*_0*kl*_, *f*_0*l*_ at household-, Si/Gun/Gu-, and Si/Do-level, respectively). The random effects parameters are each assumed to follow a normal distribution with mean 0, and variance of *u*_*0jkl*_ ~ N (0,${\sigma }_{u0}^{2}$), *v*_*0kl*_ ~ N (0, ${\sigma }_{v0}^{2}$), and *f*_*0l*_ ~ N (0, ${\sigma }_{f0}^{2}$). Since logistic regression models do not have a level 1 residual term, between-individual variance was estimated as π.^2^/3 (3.29) based on the method summarized by Goldstein et al.[35]

Two model specifications were estimated based on the general modelling structure outlined above: a null/unadjusted model, which included only an intercept term in the fixed part of the model, and a fully adjusted model. In order to develop a more detailed quantification of geographic variability in toothbrushing behavior, we calculated the variance partitioning coefficient (VPC) in the null model and fully adjusted model, which is defined as proportion of total contextual variance (excluding between-individual variance) attributable to each contextual level (household, Si/Gun/Gu, and Si/Do). Further, the proportion of variances explained by the inclusion of the individual- and Si/Gun/Gu-level characteristics in the null model compared with fully adjusted model, i.e., the proportional change in variance (PCV), was calculated at each level. Technical details for VPC and PCV are as below.

$$\mathrm{VPC}\;\mathrm{attributable}\;\mathrm{to}\;\mathrm{level}\;k\;=\;\mathrm\sigma_{k0\;}^2/\;\left(\mathrm\sigma_{u0\;}^2+\;\mathrm\sigma_{v0}^2\;+\;\mathrm\sigma_{f0}^2\right)\;\times\;100$$

$$\mathrm{PCV}\;\mathrm{at}\;\mathrm{level}\;k\;=\left(\mathrm\sigma_{k0\;\left(null\mathit\;model\right)\;\;}^2-\;\mathrm\sigma_{k0\;\left(\mathrm{fully}\;\mathrm{adjusted}\;\mathrm{model}\right)}^2\right)\;/\;\;\mathrm\sigma_{k0\;\left(\mathrm{null}\mathit\;\mathrm{model}\right)}^2\;\mathrm x\;100$$

All statistical analyses were performed using STATA version 17 (StataCorp LP, College Station, TX, USA).

### Ethical approval and consent to participate

This is the secondary analysis using the de-identified data. Therefore, it does not meet the regulatory definition of human subject research and was approved for exemption from human subjects reviews by Yonsei University Mirae Campus Bioethics Review Committee (1041849-202206-SB-099-01). Korea Disease Control and Prevention Agency (https://chs.kdca.go.kr/chs/main.do) granted permission to access the data that support the findings of this study. All methods were carried out in accordance with relevant guidelines and regulations for the present study.

## Results

### Descriptive statistics of analytic sample

Information on the density of dental facilities was not available in 40 out of 254 Si/Gun/Gus in Statistics Korea. Consequently, upon combining data from the 2017 CHS with data on two gun-level variables sourced from Statistics Korea, our original sample comprised of 192,439 observations. Following the exclusion of observation with missing values in outcome or independent variables, the final analytic sample included 182,691 individuals nested hierarchically within 100,789 households, 214 Si/Gun/Gus, and 15 Si/Dos. The missing rate from the original sample was 5.1%. Additionally, we provided the comparison of descriptive statistics from the sample before deleting observations with missing values on the density of dental facilities with those of the final analytic sample to check if missingness in the data from Statistics Korea affects national representativeness. We confirmed that there were no noteworthy differences (Table S2).

About 40% of the sample were aged 60 or older, and 35.5% had a college degree or higher. 19.1% were engaged in white collar jobs, and 31.6% in agricultural, fishing, labor, and mechanical jobs. Only 9.2% followed healthy lifestyles. 31.8% had high blood pressure or diabetes, and 27.2% were obese.

The average number of dental facilities per km^2^ in Si/Gun/Gu ranged from zero to 22, with an average of 1.8. The proportion of people washing their hands before meals ranged between 22 to 79% (Table S2).

### Fixed effects

**Fig. 2:**
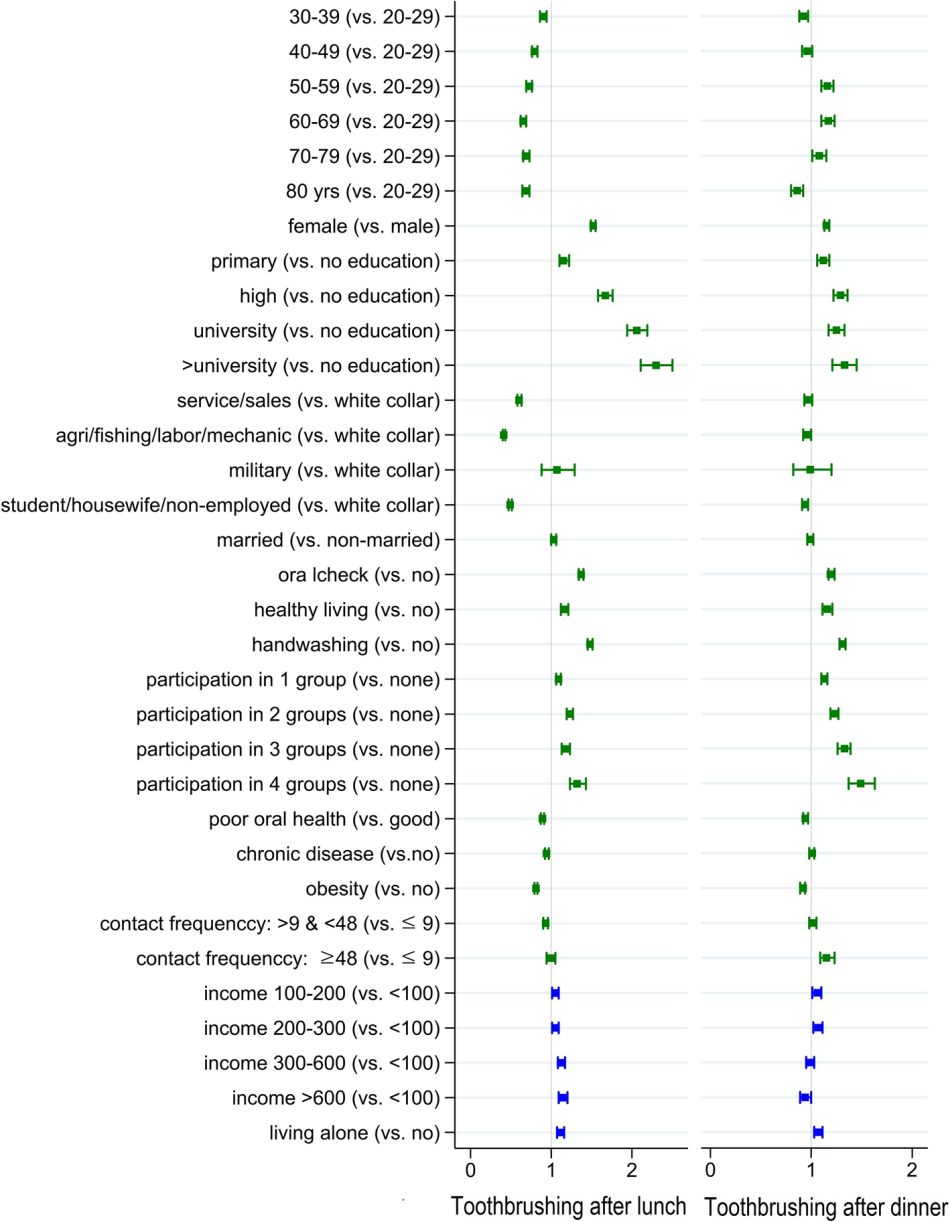
Results on the association between individual- and household-level factors and toothbrushing after lunch and dinner from four-level random intercept logistic regression

Figure 2 presents results of the fully adjusted model for toothbrushing after lunch and after dinner from four-level logistic regression.

The likelihood of brushing teeth after lunch was highest among those under 30 years, with a negative gradient based on increasing age (OR = 0.90, 0.79, 0.83, 0.65, 0.68, and 0,68 for 30–39, 40–49, 50–59, 60–68, 70–79, and ≥ 80 years old, respectively; *p* < 0.00 for all age groups). The likelihood of brushing teeth after dinner, by contrast, was lower only in the 30–39 and ≥ 80-year-old groups (OR = 0.92, 0.86 respectively; *p *< 0.00 for all) compared to those under 30 years old. The 50–79 age groups showed higher odds of brushing after dinner compared to reference group (OR = 1.16, 1.17, and 1.08 for 50–59, 60–69, and 70–79 years old, respectively; *p* < 0.00 for 50–69 years and *p* = 0.017 for 70–79 years old) (right panel in Fig. 2 and Table S3). Education level was positively associated with odds of brushing after lunch as well as brushing after dinner, but the association was stronger for toothbrushing after lunch. The odds of brushing after lunch among those with college degree or above was 2.30 (*p* < 0.00) while the odds was 1.33 for brushing after dinner in the same education group (Fig. 2 and Table S3).

Occupation showed the most distinctive patterns of the association with toothbrushing after lunch and after dinner. Compared to those working in white collar jobs, those who are engaged in service/sales, agriculture/fishing/labor/mechanics, or were student/housewife/unemployed were 0.60, 0.41, and 0.49 times less likely to brush their teeth after lunch, respectively (*p* < 0.00 for all). However, this gap significantly attenuated for brushing after dinner (OR = 0.97, 0.96, 0.94, *p* = 0.096, 0.025, and 0.001, respectively) (Fig. 2 and Table S3).

Having oral checkups, following a healthy lifestyle, handwashing before meals, and social participation all showed positive associations with toothbrushing after lunch and after dinner. Household income was positively associated with toothbrushing after lunch whereas the likelihood of brushing after dinner was only higher in income group of 100–300 (10,000 K₩) and was the lowest in the highest income group (> 600, 10,000 K₩) (Fig. 2 and Table S3).

**Fig. 3:**
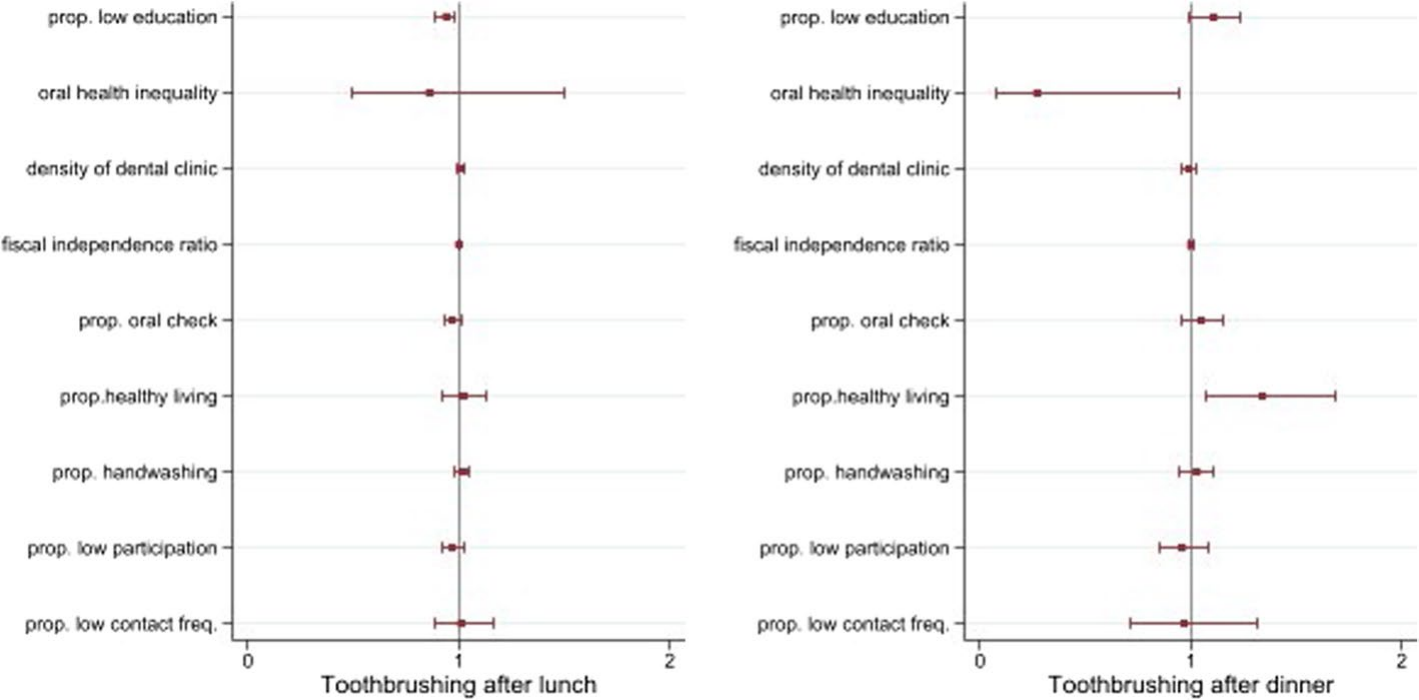
Results on the association between Si/Gun/Gu factors and toothbrushing after lunch and dinner from four-level random intercept logistic regression

Among the Si/Gun/Gu-level factors, the proportion of people with low level of education was inversely associated with the likelihood of brushing teeth after lunch while it had no significant association with brushing after dinner. Specifically, for each 10% increase in the proportion of residents with low education, the odds of brushing teeth after lunch for individuals residing in the same Si/Gun/Gu dropped by 0.94 times. Those residing in Si/Gun o/Gu with greater oral health inequalities were less likely to brush after dinner. The proportion of people practicing health lifestyle showed a positive association with brushing after dinner but no significant association with brushing after lunch. The likelihood of brushing after dinner increased by 1.34 times with every 10% increase in the proportion of people adhering to a healthy lifestyle (Fig. 3 and Table S3).

### Random effects

In the null model, which included only an intercept term, most contextual variation was attributable to households (70.2%) both for toothbrushing after lunch and after dinner, followed by Si/Gun/Gu (19.3% and 26.5% respectively). The variables included in our analyses explained the between-Si/Do variation and between-Si/Gun/Gu variation of toothbrushing after lunch by 67.4 and 57.1% respectively but did not decrease variations in toothbrushing after dinner (Table 1).

**Table 1:** Random effect from four-level random intercept logistic regression

		Toothbrushing after lunch				Toothbrushing after dinner			
		Null		Fully adjusted		Null		Fully adjusted	
		Var	SE	Var	SE	Var	SE	Var	SE
Variance	Si/Do	0.046	0.019	0.015	0.007	0.02	0.013	0.021	0.014
	Si/Gun/Gu	0.084	0.008	0.036	0.004	0.214	0.02	0.22	0.023
	Household	0.306	0.01	0.355	0.012	0.575	0.013	0.557	0.014
VPC (%)	Si/Do	10.6		3.7		2.5		2.6	
	Si/Gun/Gu	19.3		8.9		26.5		27.6	
	Household	70.2		87.4		71.1		69.8	
PCV (%)	Si/Do			67.4				-5.0	
	Si/Gun/Gu			57.1				-2.8	
	Household			-16.0				3.1	

*VPC* Variance Partitioning Coefficient, *PCV* Proportional Change in Variance

## Discussion

This is the first study comparing the differential associations between a comprehensive set of factors at multiple levels and toothbrushing behavior after lunch versus after dinner. We found a distinct pattern of association between the two. Several points that deserve discussion.

First, individual-level factors that were strongly associated with toothbrushing after lunch included younger age, higher education level, and occupation of white-collar job whereas the same variables were less strongly or not at all associated with brushing after dinner. Those in the 50–59,60–69, and 70–79 age brackets were 0.73, 0.65, and 0.68 times less likely to brush their teeth after lunch but were more likely to brush their teeth after dinner compared to the age bracket younger than 30. Similarly, those who work in service/sales or agriculture/fishery/labor/mechanical work, or were students/housewives/unemployed were 0.60, 0.41, and 0.69 times less likely to brush their teeth after lunch compared to office workers, respectively, while they did not differ with respect to the likelihood of brushing teeth after dinner compared to office workers.

The bottom four Si/Dos in toothbrushing rate after lunch both for men and women (Jeju. Gyeoungsangbuk-do, Jeollanam-do, and Jeollabuk-do in Figure S1) were in the top four with respect to the proportion of the population engaged in agriculture/fishing/labor/mechanical work, which were found to be associated with the lowest odds of toothbrushing after lunch in our analyses (Table S4). The same four Si/Dos also had the highest proportion of the population aged 50 or older, which in our analyses found to be the age group with much lower odds of toothbrushing after lunch (Table S4). These factors are assumed to make a substantial contribution to between Si/Dos variation in brushing after lunch.

It is striking that groups who were least likely to brush their teeth after lunch reported a similar (or even higher likelihood) of brushing after dinner compared to reference groups. It is possible that people compensate for their inability to brush after lunch by making up for their behavioral deficit after dinner. That is, people may still have a predisposing motivation for toothbrushing (i.e., a desire to maintain good oral hygiene), yet they are blocked by environmental circumstances, e.g., construction workers having no place to rinse on a worksite. People generally use public restrooms, however, they may not be considered sufficiently hygienic. Furthermore, since they do not have their own space (as office workers do), they cannot carry personal belongings conveniently.

It is also worth noting that there was a stronger association between education level and toothbrushing after lunch compared to toothbrushing after dinner. The effects of brushing after lunch (fresh breath, clean teeth) are observable by colleagues at work, whereas brushing after dinner is only noticed by intimate family members. It is possible that more educated individuals perform behaviors to seek approval from others compared to those with lower educational attainment.

Regarding Si/Gun/Gu characteristics, high oral health inequality within Si/Gun/Gu was strongly associated with reduced likelihood of toothbrushing after dinner. Health inequality exist because of socially determined variations in opportunities, behaviors, beliefs and exposure to a multitude of factors which influence our health. Prior studies suggest several explanations on how such health inequalities can lead to decline in overall health [36, 37]. However, further research is warranted to elucidate mechanisms at play within the field of oral health.

Finally, the factors we considered in our analyses explained 67.4% of between-Si/Do variation and 57.1% of between-Si/Gun/Gu variation in toothbrushing after lunch whereas, in toothbrushing after dinner, they rather increased the variation, which suggests that the factors generating variation at Si/Do- and Si/Gun/Gu-level are not the same between toothbrushing after lunch and after dinner. There are other factors affecting variation in toothbrushing after dinner at those contextual levels that we could not include in our analyses.

This study has some limitations. First, the cross-sectional design of the study restricts causal interpretation. However, reverse causation is not theoretically plausible in most of the variables. Second, we attempted to include variables as comprehensively as the data allowed. However, there are factors that affect toothbrushing behavior but have not been surveyed in CHS.

Finally, we restricted our analyses to certain timings of toothbrushing, namely “after lunch” and “after dinner”. While this allowed us to draw valuable insights, expanding the scope of analyses to encompass various outcomes, such as “twice a day” or “toothbrushing after breakfast”, could potentially offer a more comprehensive understanding of toothsbrushing behavior.

Despite limitations, our study provides useful insights into how factors at different levels differentially predict toothbrushing behaviors depending on the time of day and thus how we can tailor interventions to address them. Despite these limitations, our study can provide important policy implication that interventions to improve toothbrushing behavior need to be tailored depending on the time of day.

## Conclusion

Patterns in association with various factors at individual-, household- and Si/Gun/Gu-levels differed between toothbrushing after lunch versus toothbrushing after dinner, suggesting that interventions to improve toothbrushing behavior need to be tailored depending on the time of day.

### Supplementary Information

**Additional file 1: Figure S1.** Toothbrushing rate after lunch and after dinner among men and women by Si/Do (created by the author based on CHS 2015-2019). **Table S1.** Definition of independent variables. **Table S2.** Comparison of descriptive statistics of original and analytic samples. **Table S3.** Results on the association between individual-, household-and Si/Gun/Gu level factors and toothbrushing after lunch and dinner from four level random intercept logistic regression. **Table S4.** The proportion of occupational categories and age group older than 50 by Si/Do.

## Data Availability

The data that support the findings of this study are available in the Korea Disease Control and Prevention Agency repository (https://chs.kdca.go.kr/chs/main.do).
