# Supplementary material for: Factors predicting different times for brushing teeth during the day: multilevel analyses
Source: BMC Oral Health. 2023 Nov 24;23:916. doi: 10.1186/s12903-023-03555-1 (PMC10668384; doi:10.1186/s12903-023-03555-1)
Supplement: Supplementary file 1 — Additional file 1: Figure S1. Toothbrushing rate after lunch and after dinner among men and women by Si/Do (created by the author based on CHS 2015-2019). Table S1. Definition of independent variables. Table S2. Comparison of descriptive statistics of original and analytic samples. Table S3. Results on the association between individual-, household-and Si/Gun/Gu level factors and toothbrushing after lunch and dinner from four level random intercept logistic regression. Table S4. The proportion of occupational categories and age group older than 50 by Si/Do. [file 12903_2023_3555_MOESM1_ESM.docx]

Supplemental materials


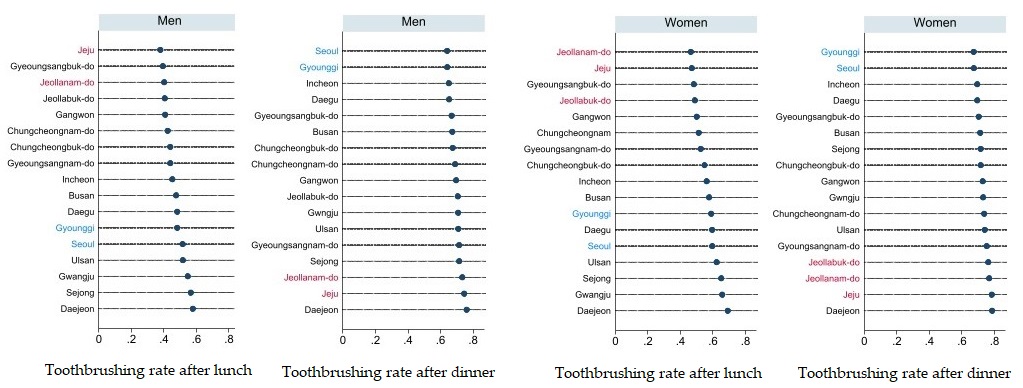


Figure S1. Toothbrushing rate after lunch and after dinner among men and women by Si/Do (created by the author based on CHS 2015-2019)

Table S1. Definition of independent variables

| 1. **Individual-level** | |
| --- | --- |
| **Demographic factors** | |
| Age | 19-29, 30-39, 40-49, 50-59, 60-69, 70-79, and ≥80 years |
| Sex | Men and women |
| **Structural factors** | |
| Education level | No education, primary graduate, high school graduate, collage graduate, higher than collage graduate |
| Occupation | White color (manager, professionals and office workers), service and sales worker, agricultural or fishing workers/laborer/machinist, military, and student/housewives/non-employed. |
| Marital status | Married and non-married (divorced, separated, widowed, and single) |
| **Behavioral factors** | |
| Oral health checkup | Yes (having no particular dental problem, but getting oral health checked in the past year) or no |
| Healthy living | Yes (meeting all three of the following) and no   - No smoking: has never smoked or smoked in the past but currently does not smoke - No drinking alcohol: has never drunken or have drunken in the past but currently does not drink - Walking: walked for than 30 minutes more than five days last week |
| Handwashing before meal | Yes (always or frequently washed hand before meal last week) and no (sometimes or rarely) |
| Participation in activities (score range: 0~4) | The number of participating activities among four: religion, socializing (alumni association, clan association etc.), recreational, and volunteering activities. |
| **Biological factors** | |
| Self-rated oral health | Good (very good, good, or not good or not bad for the question: “How do you generally rate your oral health”) and poor (poor and very poor) |
| Chronic disease | Yes (diagnosed with hypertension or diabetes) or no |
| Obesity | Yes (BMI (kg/m^2^) ≥25) or no |
| **Moderating factor (social capital)** | |
| Contact frequency | Frequency of meeting or contacting family members or relatives or neighbors or friends per month: ≤9, >9 & <48, and ≥48 |
| **Household-level** | |
| Income | Household monthly income (unit: 10,000 K₩): <100, ≥100 &<200, ≥200 &<3,000, ≥300 &<600, and ≥600 |
| Living alone | Yes or no |
| **Si/Gun/Gu-level** | |
| **Structural factors** | |
| Proportion of the population with low levels of education^§^ | Proportion of non-educated or primary graduate. |
| Disparity in oral health | Standard deviation of self-rated oral health score (1: very poor ~ 5: very good) |
| Dental facility density | The number of dental facilities (clinics and hospitals)/km^2^ |
| Fiscal independence | Ratio of locally-generated revenue of total revenue |
| **Behavioral factors** | |
| Proportion of people who had oral checkup^§^ | The proportion of people who have had no particular dental problem but have had an oral checkup in the past year |
| Proportion of people who practice healthy lifestyle^§^ | The proportion of people who follow healthy lifestyle defined above |
| Proportion of people who wash hands before meal ^§^ | Proportion of people who washed hands before meal always or frequently in the past week. |
| Proportion of people who had low contact frequency^§^ | Proportion of people meeting or contacting with family member or relatives or neighbors or friends fewer than nine times per month |
| **Moderating factor (social capital)** | |
| Average number of participating activities | Proportion of people who do not participate in any of the following four activities: religion, socializing, recreational, and volunteering activities. |

§: Proportion (0~1) was multiplied by 10 for convenience of interpretation. As a result, coefficient in the regression is interpreted as the change in the probability of toothbrushing by the increase of one point.

Table S2. Comparison of descriptive statistics of original and analytic samples.

|  | Total | | | Sample before merging  (N=228,381) | | | Analytic sample  (N = 182,691) | |
| --- | --- | --- | --- | --- | --- | --- | --- | --- |
| Individual level variables | | | | | | | | |
| Age (years) | 19-29 | | | 24328 (10.7%) | | | 18709 (10.2%) | |
|  | 30-39 | | | 29887 (13.1%) | | | 23274 (12.7%) | |
|  | 40-49 | | | 39484 (17.3%) | | | 31230 (17.1%) | |
|  | 50-59 | | | 45144 (19.8%) | | | 36939 (20.2%) | |
|  | 60-69 | | | 40748 (17.8%) | | | 34581 (18.9%) | |
|  | 70-79 | | | 33783 (14.8%) | | | 27363 (15.0%) | |
|  | ≥ 80 | | | 15007 (6.6%) | | | 10595 (5.8%) | |
| Sex | Male | | | 102484 (44.9%) | | | 83980 (46.0%) | |
|  | Female | | | 125897 (55.1%) | | | 98711 (54.0%) | |
| Education level | No education | | | 15256 (6.7%) | | | 10468 (5.7%) | |
|  | Primary graduate | | | 39309 (17.2%) | | | 32835 (18.0%) | |
|  | High school graduate | | | 90596 (39.7%) | | | 74619 (40.8%) | |
|  | Collage graduate | | | 75062 (32.9%) | | | 58618 (32.1%) | |
|  | >Collage graduate | | | 7899 (3.5%) | | | 6151 (3.4%) | |
|  | Missing | | | 259(0.1%) | | |  | |
| Occupation | White collar | | | 44960 (19.7%) | | | 34969 (19.1%) | |
|  | Sales workers | | | 29270 (12.8%) | | | 23611 (12.9%) | |
|  | Agri/fishing/labor/mechanic | | | 69448 (30.4%) | | | 57734 (31.6%) | |
|  | Military | | | 715 (0.3%) | | | 618 (0.3%) | |
|  | Student/housewife/non-employed | | | 83721 (36.7%) | | | 65759 (36.0%) | |
|  | Missing | | | 267 (0.1%) | | |  | |
| Marital status | Non-married | | | 74196 (32.5%) | | | 57610 (31.5%) | |
|  | Married | | | 153931 (67.4%) | | | 125081 (68.5%) | |
|  | Missing | | | 254 (0.1%) | | |  | |
| Oral health checkup | No | | | 145049 (63.5%) | | | 117133 (64.1%) | |
|  | Yes | | | 83286 (36.5%) | | | 65558 (35.9%) | |
|  | Missing | | | 46 (0.0%) | | |  | |
| Healthy lifestyle | No | | | 208149 (91.1%) | | | 165895 (90.8%) | |
|  | Yes | | | 20136 (8.8%) | | | 16796 (9.2%) | |
|  | Missing | | | 96 (0.0%) | | |  | |
| Handwashing before meal | No | | | 100,934 (44.2%) | | | 80097 (43.8%) | |
|  | Yes | | | 127,428 (55.8%) | | | 102594 (56.2%) | |
|  | Missing | | | 19 (0.01%) | | |  | |
| Participation in activities | 0 | | | 67000 (29.3%) | | | 51752 (28.3%) | |
|  | 1 | | | 84598 (37.0%) | | | 68457 (37.5%) | |
|  | 2 | | | 54327 (23.8%) | | | 44180 (24.2%) | |
|  | 3 | | | 17537 (7.7%) | | | 14371 (7.9%) | |
|  | 4 | | | 4869 (2.1%) | | | 3931 (2.2%) | |
|  | Missing | | | 50 (0.0%) | | |  | |
| Self-rated oral health | Not poor | | | 143587 (62.9%) | | | 115277 (63.1%) | |
|  | Poor | | | 84775 (37.1%) | | | 67414 (36.9%) | |
|  | Missing | | | 19 (0.0%) | | |  | |
| Chronic disease | No | | | 156138 (68.4%) | | | 124575 (68.2%) | |
|  | Yes | | | 72165 (31.6%) | | | 58116 (31.8%) | |
|  | Missing | | | 78 (0.0%) | | |  | |
| Obesity | No | | | 159722 (69.9%) | | | 132982 (72.8%) | |
|  | Yes | | | 60056 (26.3%) | | | 49709 (27.2%) | |
|  | Missing | | | 8603 (3.8%) | | |  | |
| Contact frequency | ≤9 | | | 30711(13.5%) | | | 32054 (17.5%) | |
|  | >9 & <48 | | | 185335(81.2%) | | | 144719 (79.2%) | |
|  | ≥48 | | | 12324 (5.4%) | | | 5919 (3.2%) | |
|  | Missing | | | 11 (0.0%) | | |  | |
| Household-level variables | | | | | | | | |
|  |  | | | | | | 100,789 | |
| Income  (10,000 K₩) | <100 | | | 45818 (20.1%) | | | 27099 (26.9%) | |
|  | ≥100 &<200 | | | 35909 (15.7%) | | | 18340 (18.2%) | |
|  | ≥200 &<300 | | | 39130 (17.1%) | | | 17603 (17.5%) | |
|  | ≥300 &<600 | | | 78746 (34.5%) | | | 29288 (29.1%) | |
|  | ≥600 | | | 26700 (11.7%) | | | 8459 (8.4%) | |
|  | Missing | | | 2078 (0.9%) | | |  | |
| Living alone | No | | | 195629 (85.7%) | | | 75112 (74.5%) | |
|  | Yes | | | 32752 (14.3%) | | | 25677 (25.5%) | |
|  | Sample before merging | | | | Analytic sample | | | |
| Si/Gun/Gu-level variables | Mean (SD) | Min | Max | | Mean (SD) | Min | | Max |
| Prop. low education (0~10) | 2.6(1.5) | 0.2 | 6.5 | | 2.6 (1.5) | 0.2 | | 6.5 |
| Oral health inequality | 0.95 (0.1) | 0.8 | 1.1 | | 0.95 (0.1) | 0.8 | | 1.1 |
| Density of dental facilities (per km^2^) | 1.80 (3.3) | 0.0 | 21.9 | | 1.80 (3.4) | 0.0 | | 21.9 |
| Fiscal independence ratio (%) | 26.0 (12.7) | 8.6 | 68.5 | | 26.3 (12.7) | 8.6 | | 68.5 |
| Prop. oral check-up (0~10) | 3.5(1.3) | 0.5 | 6.8 | | 3.5 (1.3) | 0.5 | | 6.8 |
| Prop. practice of healthy lifestyle (0~10) | 0.9 (0.3) | 0.3 | 2.2 | | 0.9 (0.3) | 0.3 | | 2.2 |
| Prop. handwashing before meal (0~10) | 5.6 (0.8) | 2.2 | 7.9 | | 5.6 (0.9) | 2.2 | | 7.9 |
| Prop. low contact frequency (0~10) | 0.6 (0.4) | 0.0 | 1.9 | | 0.6 (0.4) | 0.0 | | 1.7 |
| Prop. low participation (0~10) | 2.9 (0.7) | 1.5 | 5.8 | | 2.9 (0.7) | 1.5 | | 5.8 |

Table S3. Results on the association between individual-, household-and Si/Gun/Gu level factors and toothbrushing after lunch and dinner from four level random intercept logistic regression.

|  | Toothbrushing after lunch | | Toothbrushing after dinner | |
| --- | --- | --- | --- | --- |
|  | OR | *p* | OR | *p* |
| Individual-level |  |  |  |  |
| Age (ref= <30) |  |  |  |  |
| 30-39 | 0.90 | 0.000 | 0.92 | 0.002 |
| 40-49 | 0.79 | 0.000 | 0.96 | 0.126 |
| 50-59 | 0.73 | 0.000 | 1.16 | 0.000 |
| 60-69 | 0.65 | 0.000 | 1.17 | 0.000 |
| 70-79 | 0.68 | 0.000 | 1.08 | 0.017 |
| ≥80 | 0.68 | 0.000 | 0.86 | 0.000 |
| Sex (ref=male) |  |  |  |  |
| female | 1.52 | 0.000 | 1.15 | 0.000 |
| Education (ref=no education) |  |  |  |  |
| primary graduate | 1.15 | 0.000 | 1.12 | 0.000 |
| high school graduate | 1.67 | 0.000 | 1.29 | 0.000 |
| university graduate | 2.06 | 0.000 | 1.25 | 0.000 |
| >university | 2.30 | 0.000 | 1.33 | 0.000 |
| Job (ref=white collar) |  |  |  |  |
| service/sales | 0.60 | 0.000 | 0.97 | 0.096 |
| agri/fishing/labor/mechanic | 0.41 | 0.000 | 0.96 | 0.025 |
| military | 1.07 | 0.494 | 0.99 | 0.912 |
| student/housewife/unemployed | 0.49 | 0.000 | 0.94 | 0.001 |
| Marital status (ref=unmarried) |  |  |  |  |
| married | 1.03 | 0.054 | 0.99 | 0.454 |
| Frequency of eating breakfast |  |  |  |  |
| Oral checkup (ref=no) |  |  |  |  |
| yes | 1.37 | 0 | 1.19 | 0.000 |
| Healthy living (ref=no) |  |  |  |  |
| yes | 1.16 | 0 | 1.16 | 0.000 |
| Handwashing (ref=hardly) |  |  |  |  |
| always/frequently | 1.48 | 0 | 1.31 | 0.000 |
| Poor SR oral health (ref=no) |  |  |  |  |
| yes | 0.89 | 0.000 | 0.94 | 0.000 |
| Chronic disease(ref=no) |  |  |  |  |
| yes | 0.94 | 0.000 | 1.01 | 0.637 |
| Obesity(ref=no) |  |  |  |  |
| yes | 0.81 | 0.000 | 0.92 | 0.000 |
| Contact frequency (ref= ≤9) |  |  |  |  |
| >9 & <48 | 0.93 | 0.000 | 1.01 | 0.510 |
| ≥ 48 | 1.00 | 0.893 | 1.15 | 0.000 |
| N of participation group (ref=0) |  |  |  |  |
| 1 | 1.09 | 0.000 | 1.13 | 0.000 |
| 2 | 1.23 | 0.000 | 1.23 | 0.000 |
| 3 | 1.18 | 0.000 | 1.33 | 0.000 |
| 4 | 1.32 | 0.000 | 1.49 | 0.000 |
| Household level |  |  |  |  |
| Monthly income (ref: <10,000K₩^†^) |  |  |  |  |
| 100-200 | 1.05 | 0.009 | 1.06 | 0.008 |
| 200-300 | 1.05 | 0.024 | 1.07 | 0.003 |
| 300-600 | 1.13 | 0.000 | 0.99 | 0.538 |
| >600 | 1.14 | 0.000 | 0.94 | 0.034 |
| Living alone (ref=no) |  |  |  |  |
| yes | 1.11 | 0.000 | 1.07 | 0.001 |
| Si/Gun/Gu level |  |  |  |  |
| Prop. poorly educated (0~10) | 0.94 | 0.009 | 1.11 | 0.062 |
| Oral health inequality | 0.86 | 0.588 | 0.27 | 0.041 |
| Density of dental facilities^§^ | 1.01 | 0.447 | 0.99 | 0.653 |
| Fiscal self-reliance ratio (%) | 1.00 | 0.792 | 1.00 | 0.556 |
| Prop. people oral check-up (0~10) | 0.97 | 0.089 | 1.05 | 0.305 |
| Prop. practice of healthy living (0~10) | 1.02 | 0.712 | 1.34 | 0.011 |
| Prop. handwashing (0~10) | 1.02 | 0.313 | 1.03 | 0.467 |
| Prop. low contact frequency (0~10) | 0.97 | 0.342 | 0.96 | 0.508 |
| Prop. low participation (0~10) | 1.01 | 0.838 | 0.97 | 0.830 |

Table S4. The proportion of occupational categories and age group older than 50 by Si/Do

| Si/do | Occupation | | | | | | Age |
| --- | --- | --- | --- | --- | --- | --- | --- |
|  | Office work | Service/  sales | Agriculture/fishery/  labor/mechanical work | Military | Students/housewives/  the unemployed | | ≥50 years |
| Seoul Si | 32.5 | 14.7 | 14.4 | 0.1 | | 38.3 | 48.0 |
| Busan Si | 21.0 | 15.3 | 20.8 | 0.0 | | 42.8 | 56.1 |
| Daegu Si | 20.4 | 13.1 | 23.3 | 0.1 | | 43.1 | 52.2 |
| Incheon Si | 19.8 | 15.1 | 28.7 | 0.1 | | 36.2 | 54.7 |
| Gwangju Si | 24.9 | 16.5 | 20.8 | 0.1 | | 37.7 | 49.6 |
| Daejeon Si | 26.3 | 14.8 | 18.1 | 0.2 | | 40.6 | 49.4 |
| Ulsan Si | 22.1 | 13.2 | 26.0 | 0.0 | | 38.7 | 46.8 |
| Sejong Si | 32.8 | 10.5 | 20.7 | 0.7 | | 35.4 | 40.8 |
| Gyeonggi-do | 28.2 | 13.0 | 21.4 | 0.4 | | 37.0 | 47.1 |
| Gangwon-do | 14.0 | 14.4 | 34.9 | 1.8 | | 34.9 | 67.0 |
| Chungcheongbuk-do | 16.3 | 12.7 | 36.9 | 0.3 | | 33.8 | 61.7 |
| Chungcheongnam-do | 14.7 | 11.9 | 39.5 | 0.4 | | 33.4 | 64.6 |
| **Jeollabuk-do** | 11.4 | 9.7 | **39.1** | 0.0 | | 39.7 | **72.1** |
| **Jeollanam-do** | 11.5 | 10.3 | **45.4** | 0.1 | | 32.7 | **73.4** |
| **Gyeongsangbuk-do** | 11.7 | 10.5 | **41.3** | 0.1 | | 36.4 | **69.7** |
| Gyeongsangnam-do | 15.3 | 11.5 | 36.5 | 0.3 | | 36.5 | 64.6 |
| **Jeju-do** | 15.1 | 17.0 | **41.2** | 0.0 | | 26.6 | **59.9** |
